# Supplementary material for: Demonstration of intracellular real-time molecular quantification via FRET-enhanced optical microcavity
Source: Nat Commun. 2022 Nov 5;13:6685. doi: 10.1038/s41467-022-34547-4 (PMC9637138; doi:10.1038/s41467-022-34547-4)
Supplement: Supplementary file 1 — Supplementary Information [file 41467_2022_34547_MOESM1_ESM.pdf]

Supplementary Information for

Demonstration of intracellular real-time molecular quantification via FRET-enhanced optical microcavity

Yaping Wang[1] †, Marion C Lang[1,2] †, Jinsong Lu[1]†, Mingqian Suo[1], Mengcong Du[1], Yubin Hou[3,4,5,6], Xiu-Hong Wang[1, 4,5,6]* and Pu Wang[3,4,5,6]

[1] Laboratory for Biomedical Photonics, Institute of Laser Engineering, Faculty of Materials and Manufacturing, Beijing University of Technology; Beijing 100124 China

[2] Currently: Email: Marion.lang@zeiss.com, Carl Zeiss Microscopy GmbH ZEISS Group, Kistlerhofstr.75 81379 Munich, Germany

[3] Laboratory for Advanced Laser Technology and Applications, Faculty of Materials and Manufacturing, Beijing University of Technology; Beijing 100124 China

[4] Key Laboratory of Trans-scale Laser Manufacturing Technology, Ministry of Education, China.

[5] Beijing Engineering Research Center of Laser Technology; Beijing, China

[6] Beijing Colleges and Universities Engineering Research Center of Advanced Laser Manufacturing, Beijing, China

† These authors contributed equally.

*Corresponding author:

Xiu-Hong Wang, Ph.D.

Beijing University of Technology

100 Pingleyuan, Chaoyang District

Beijing, 100124, China

Tel: 00-86-10-67391927

Email: wxh2012@bjut.edu.cn

Contents

1. **Supplementary figures:**
   Fig. S1 and legend
   Fig. S2 and legend
   Fig. S3 and legend
   Fig. S4 and legend
   Fig. S5 and legend
   Fig. S6 and legend
   Fig. S7 and legend
   Fig. S8 and legend
   Fig. S9 and legend
   Fig. S10 and legend
   Fig. S11 and legend
   Fig. S12 and legend
   Fig. S13 and legend
   Fig. S14 and legend
   Fig. S15 and legend

2. **Supplementary tables:**
   Table S1
   Table S2
   Table S3
   Table S4

3. **Theoretical model for explanation of the sensing mechanism**

4. **Quantitative analysis of wavelength gap ($\Delta\lambda$)—concentration relationship**

5. **Supplementary methods**

6. **References**

1. Supplementary figures

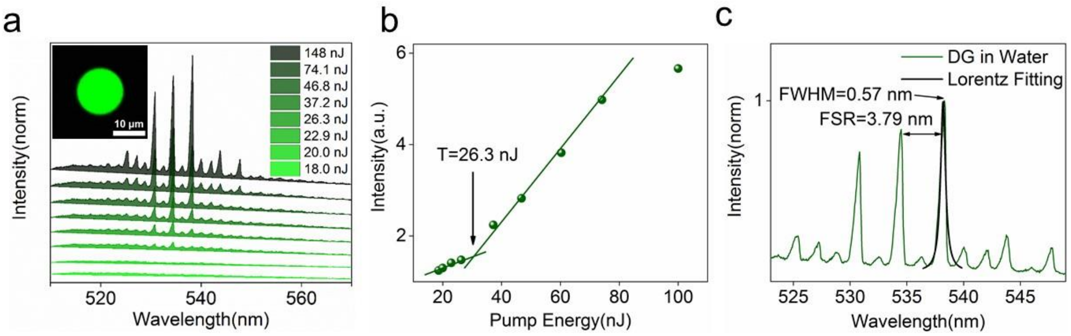

Fig. S1. WGM lasing from a DG dye doped microsphere. a, A typical WGM spectrum of a single DG bead in ultrapure water (ddH_2O) after overcoming the lasing threshold. A DG microsphere was embedded in ddH_2O and excited with a 473 nm nano-second pulsed laser. The plot shows the lasing spectra for increasing pumping fluence. The inset shows the confocal image of the DG microbead. The center peak consistently remains at the maximum of the spectral envelope under optimized excitation conditions, so it can be easily identified without ambiguousness. Only under saturated pumping conditions, we observed that the position of the maximum peak does not coincide with the maximum of the spectral envelope any more. **b**, Lasing threshold of a DG microsphere in ddH_2O. The threshold is 26.3nJ. Above the threshold, there is a linear correlation between pump energy and lasing intensity. Experiments were conducted 3 times independently with similar results and as shown are representative. **c**, The free spectral range (FSR) measured from the lasing spectrum is 3.79 nm, which is in good agreement with the estimated value of 3.81 nm (calculated using FSR $=\lambda^2/n\pi D$, D=15.25μm). The Q factor is around 10^3.

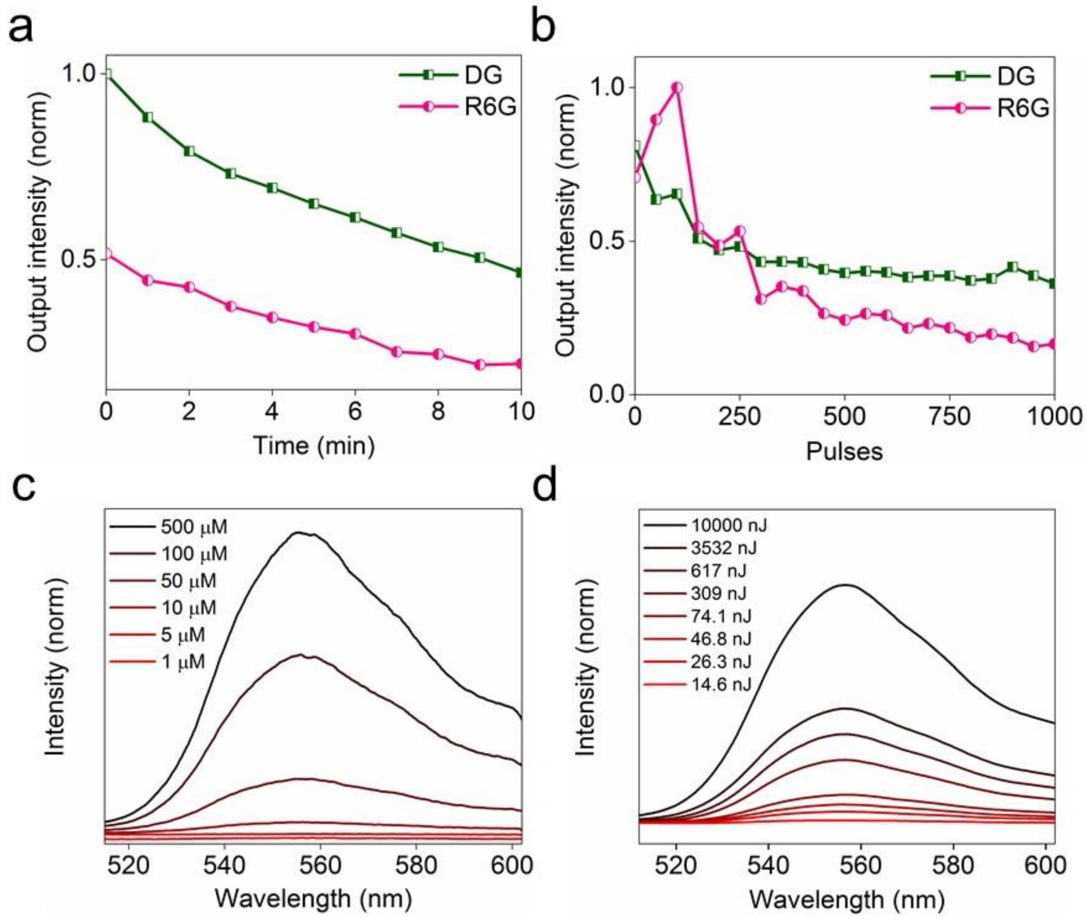

Fig. S2. a & b, Time-dependent and pulse count-dependent lasing of a DG microsphere. The decreased lasing intensity over time and pulse count, respectively, indicate the occurrence of photo bleaching. **c & d, Fluorescence emission of R6G.** When the DG microsphere is replaced with a non-fluorescent sodalime microsphere, with similar dimension and refractive index (*D*: 15μm *n*=1.59), and excited with a 473nm laser, only fluorescence emission instead of lasing emission was obtained. **c**, A sodalime microsphere was embedded in various concentrations of R6G and pumped with a 473nm pulsed laser, 617nJ. **d**, A sodalime microsphere was embedded in 50μM R6G and pumped with a 473nm laser with various powers from 14.6nJ to 10000nJ. The experiments were repeated at least three times independently with similar results.

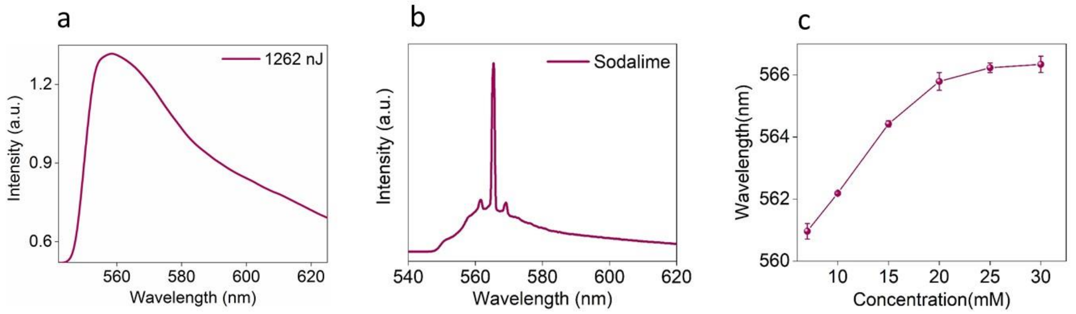

Fig. S3. Emission spectrum of R6G excited by a 532nm laser under different conditions. a. Fluorescence emission of R6G. A sodalime microsphere with same cavity size and refractive index (*n*) was embedded in 50μM R6G solution and excited with a 532nm nano-second pulsed laser (1262nJ). We observed fluorescence emission with a maximum at 558nm rather than lasing. **b.** Laser emission was observed when the R6G concentration was increased to 5mM and the pumping energy was increased to 1.26mJ. **c.** R6G emission center wavelength-concentration curve. Data are presented as mean values +/- SD (*n*=3).

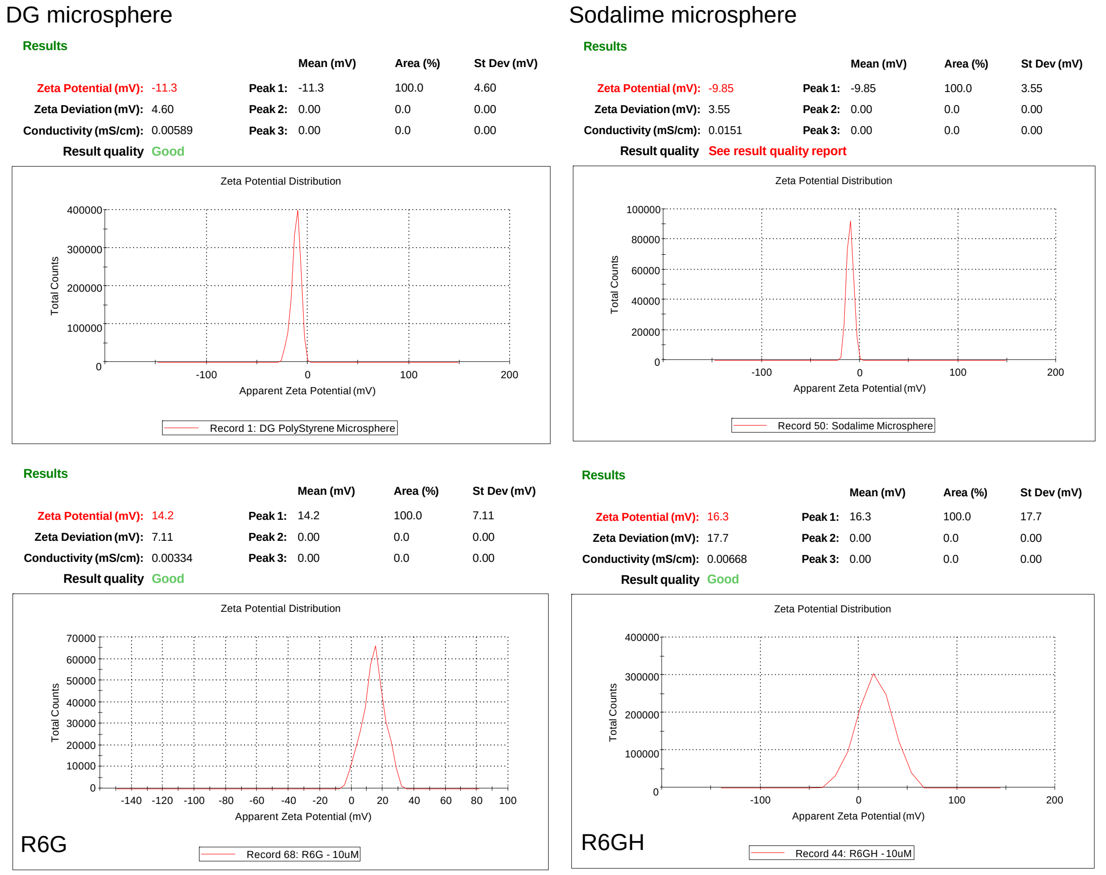

Fig. S4. Charging properties of the microspheres, R6G and R6GH molecules. Data were measured on a Zetasizer Nano ZS (Malvern Panalytical)

The measured zeta potentials of DG doped microsphere, sodalime microsphere, R6G and R6GH are:

DG doped microsphere: -11.3mV
Sodalime microsphere: -9.85mV
R6G molecules: +14.2mV
R6GH molecules: +16.3mV

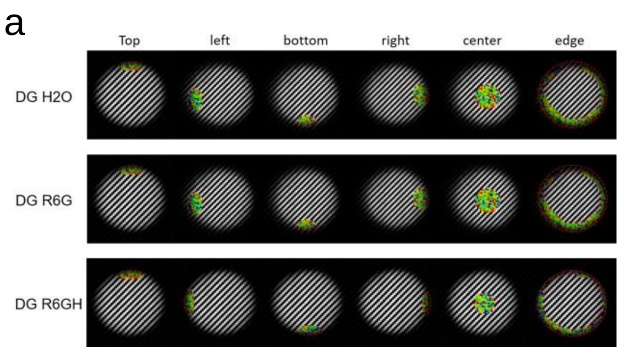

b

Fluorescence lifetime of DG at different ROI

ROI	τ (ns) DG-H2O	τ (ns) DG-R6G	τ (ns) DG-R6GH
top	4.08	1.99	4.14
left	4.06	2.21	4.03
bottom	4.15	2.42	4.15
right	4.16	2.39	3.95
center	4.17	4.10	4.00
edge	4.11	1.69	4.04

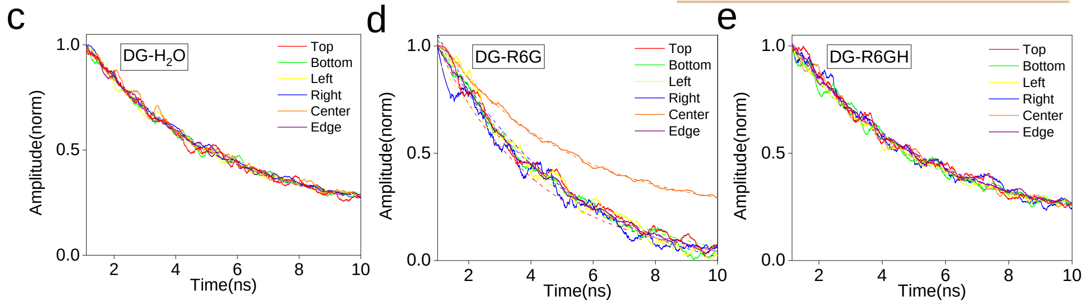

Fig. S5. Time-resolved fluorescence decay of DG molecules in the presence of R6G. **a.** Regions of interest (ROI) selection. The black-white striped areas represent the cross-section of the DG doped microsphere. DG doped microspheres were ingrained in H2O, 100µM R6G and R6GH respectively. Fluorescence decay was recorded using TCSPC as described in the experimental section via acquisition of 300000 photons. **b.** Fluorescence lifetime of each ROI (top, bottom, left, right, center and edge) under different conditions. Data in the table of each ROI are mean values of 5 repeated measurements. Since FRET efficiency is directly dependent on the donor-acceptor-A distance and the orientation of the donor and acceptor molecules, the different lifetimes obtained from the measurements indicate that the orientation of R6G molecules and the distances between DG and R6G molecules differ in the three conditions. **c,d &e**. Time resolved fluorescence decay curves of DG microspheres in H2O, R6G and R6GH.

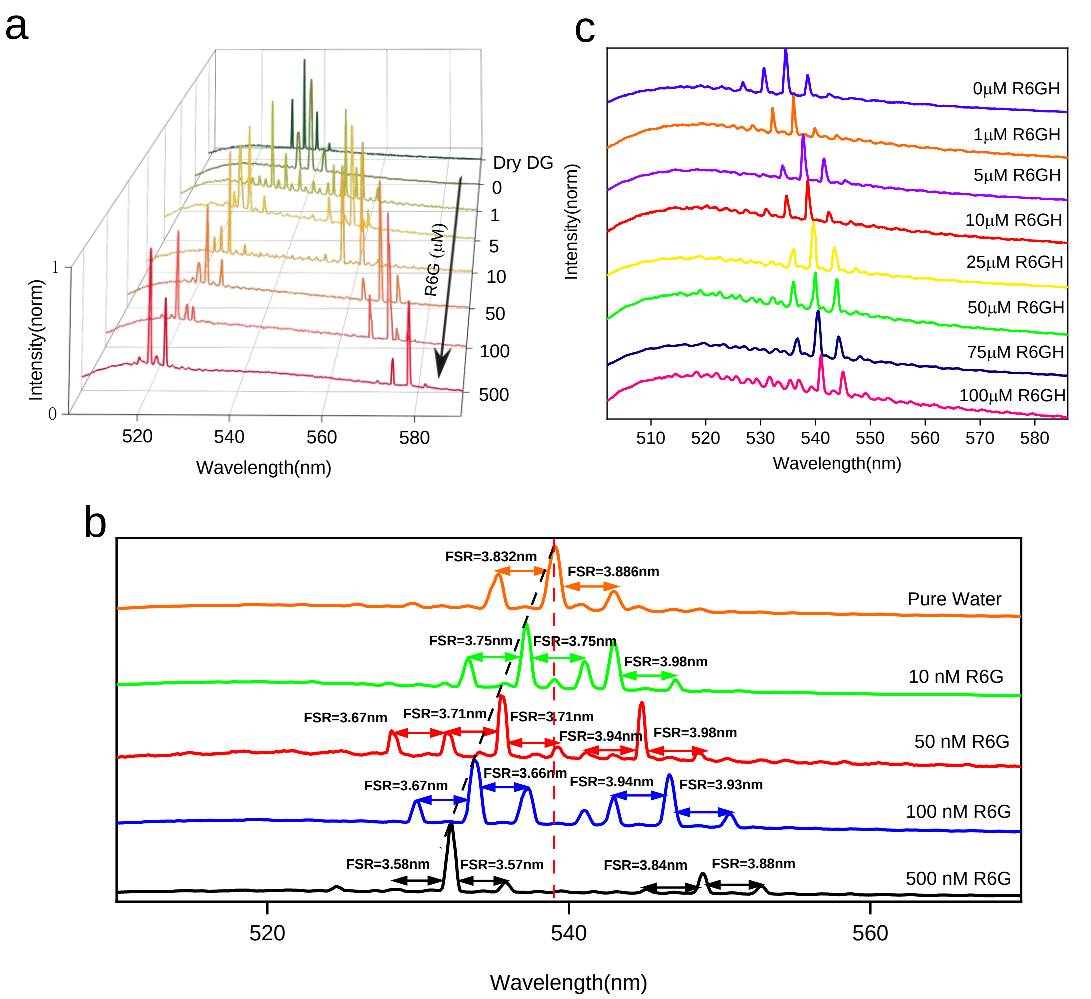

Fig. S6. **Full spectra of DG and R6G lasing**. **a&b**. Dual lasing of DG and R6G in the FRET-WGM system. DG microspheres in various concentrations of R6G (**a.** 1-500 µM; **b**. 0-500 nM; solvent is ddH2O) and pumped a with 473nm laser. At very low concentrations, such as 10nM and 50nM, we used FSR to help assigning the peaks. The FSR of R6G is always larger than that of DG, which provides a basis for us to distinguish the two spectral envelops at very low concentration. For example, at 50nM concentration, the FSRs of DG is 3.715nm, while the FSR of R6G is 3.981nm. By measuring the FSRs of two adjacent modes on the spectrum, peaks 1, 2, 3, 4 are assigned to DG lines; while peaks 5, 6 and 7 are assigned to R6G lines. Similarly, we assigned the peaks at concentration of 10nM. **c**. DG lasing in the Non-FRET-WGM system. DG microspheres in various concentrations of R6GH (solvent is 90%H_2O+10%EtOH) and pumped with a 473nm laser.

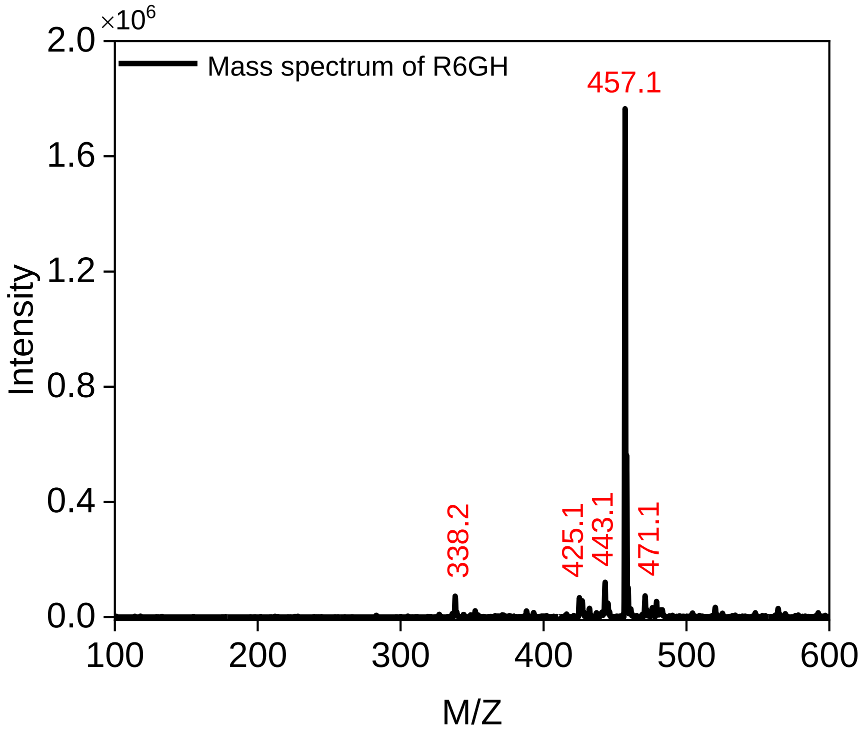

Fig. S7. Mass spectrum of R6GH. ESI ion source; positive ion mode. The major peak is M/Z (+) = 457

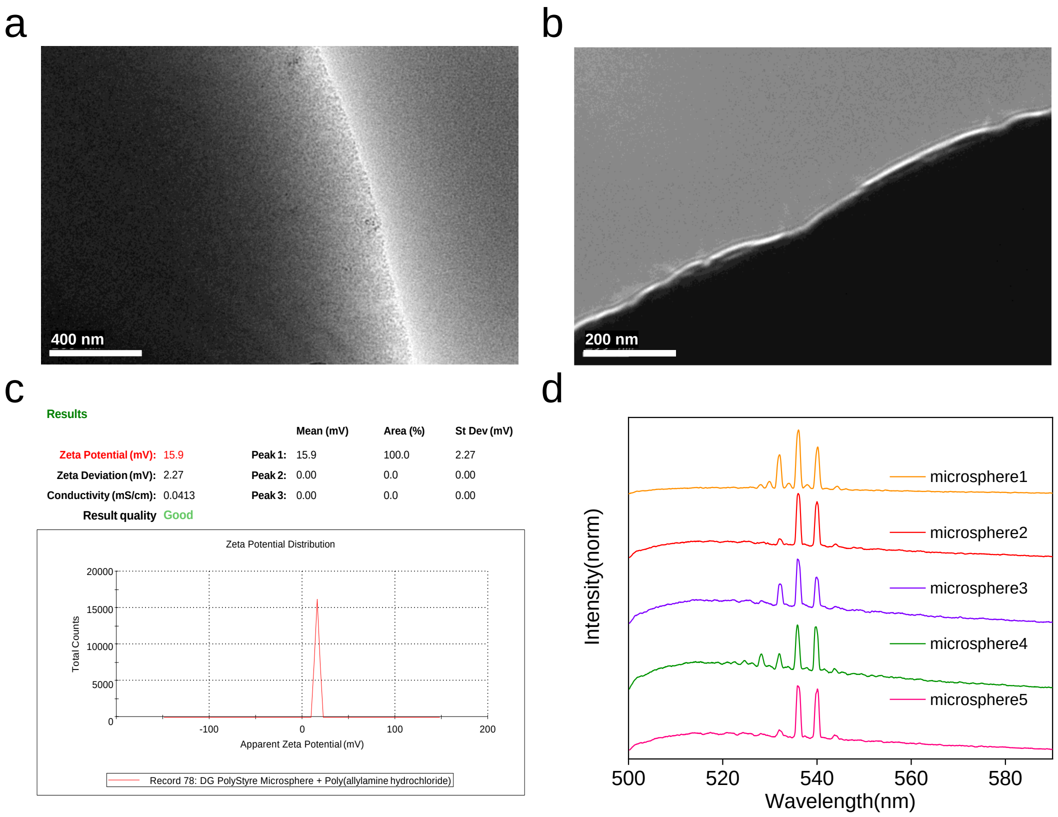

Fig. S8. Altering the surface charge of the microspheres to abolish FRET. a & b. TEM images show the morphology of a microsphere prior (**a**) and post (**b**) PAH coating. Each experiment was repeated independently three times with similar results. **c.** Zeta potential of the PAH modified microsphere is 15.9mV. **d.** DG lasing spectra after surface charge alteration. A PAH-modified DG microsphere was embedded in 50µM R6G solution and pumped with a 473nm laser. Five microspheres were randomly selected and the lasing spectra are shown. Only DG lasing, with a CWL at 535.8nm, rather than DG and R6G dual lasing were observed indicating that the FRET effect successfully was abolished by changing the charging of the DG microspheres.

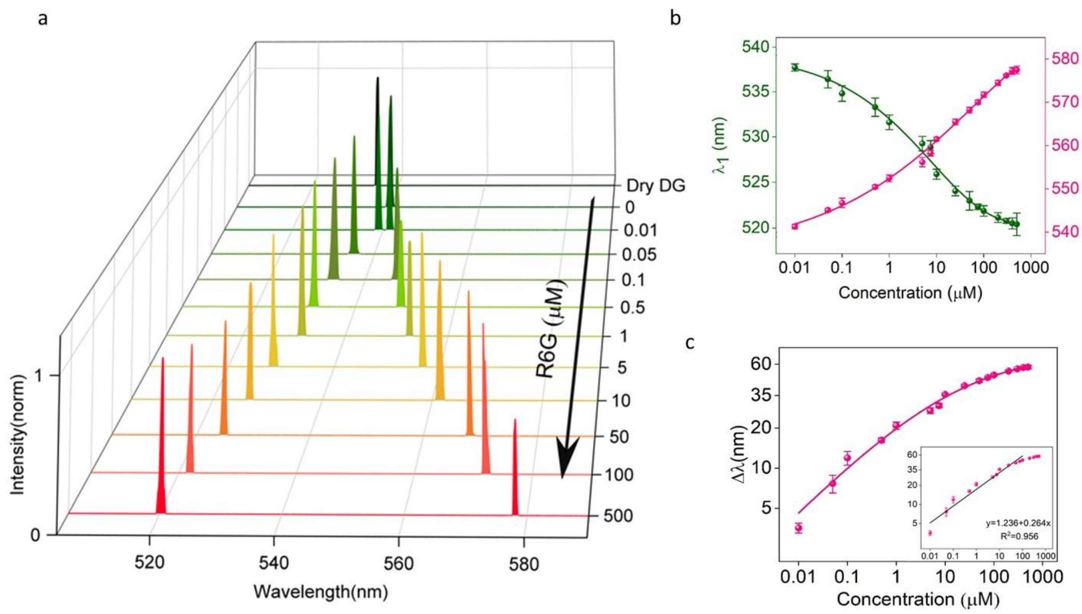

Fig. S9. FRET-WGM sensing performance using a 2% DG doped microcavity. a. Dose-dependent lasing spectra of DG and R6G. Only the CWLs of DG and R6G are shown. **b**. Dose-dependent CWL curves of DG and R6G (log scale). FSR and linewidth. Data are presented as mean values +/- SD (*n*=3). **c.** Dose-dependent $\Delta\lambda$ (λ_{R6G}-λ_{DG}) curve. The inset shows linear fit to the curve. Data are presented as mean values +/- SD (*n*=3).

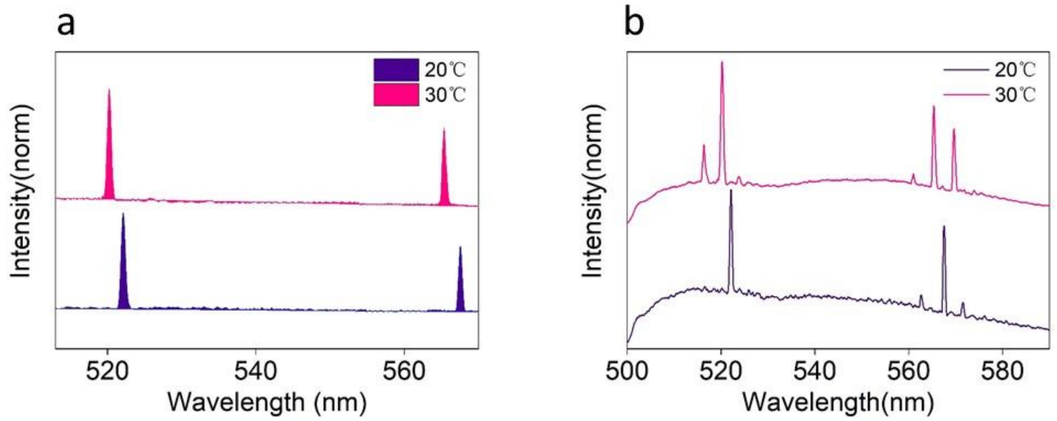

Fig. S10. Effect of temperature on FRET-WGM sensing. **a.** The CWL of DG and R6G lasing at a temperature of 20°C (blue) and 30°C (pink), respectively. A DG-doped microsphere was ingrained in 75μM R6G solution and pumped with a 473nm laser at 20°C and 30°C, respectively. Higher temperature resulted in a shift of the resonance wavelengths of DG and R6G toward shorter wavelengths with $\Delta\lambda$ (λ_{R6G}-λ_{DG}) at 30°C of 45.17nm, and of 45.41nm at 20°C. **b.** Full spectra of the measurement shown in (b). The experiment was repeated three times independently with similar results.

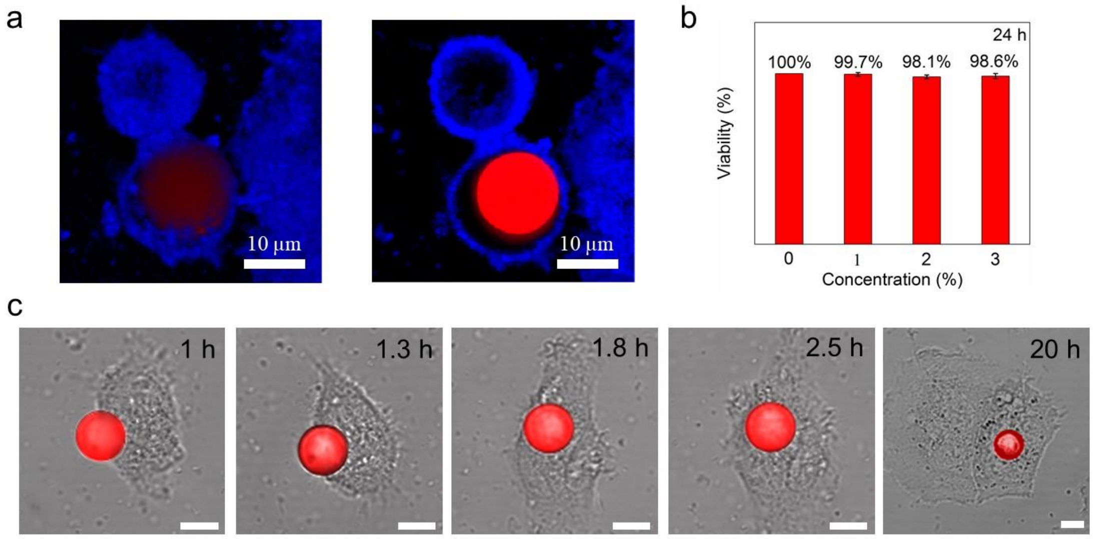

Fig. S11. Cells harboring DG microspheres grow and divide as usual. **a**, Left: Laser scanning confocal microscopic (LSCM) 3D imaging of two cells, one harboring a DG microbead and the other one not. Right: cross section of the LSCM image showing that the DG microbead is indeed inside the cell instead of sitting on the top of the cell. Each experiment was repeated three times independently with similar results. **b**, Cell viability after incubation with various concentrations of DG microbeads for 24h. Data are presented as mean values +/- SD (*n*=3). **c**, Time-lapse imaging of a DG micro-bead entering a cell. After 2.5h, the cell completely incorporated the microbead. After 20h, the mother cell divides into two daughter cells and the bead stays in one of the daughter cell. The data indicate that DG micro-beads can easily enter cells via endocytosis and stay in the cells without disrupting cell growth or division. Each experiment was repeated three times independently with similar results. Scale bars 10 μm.

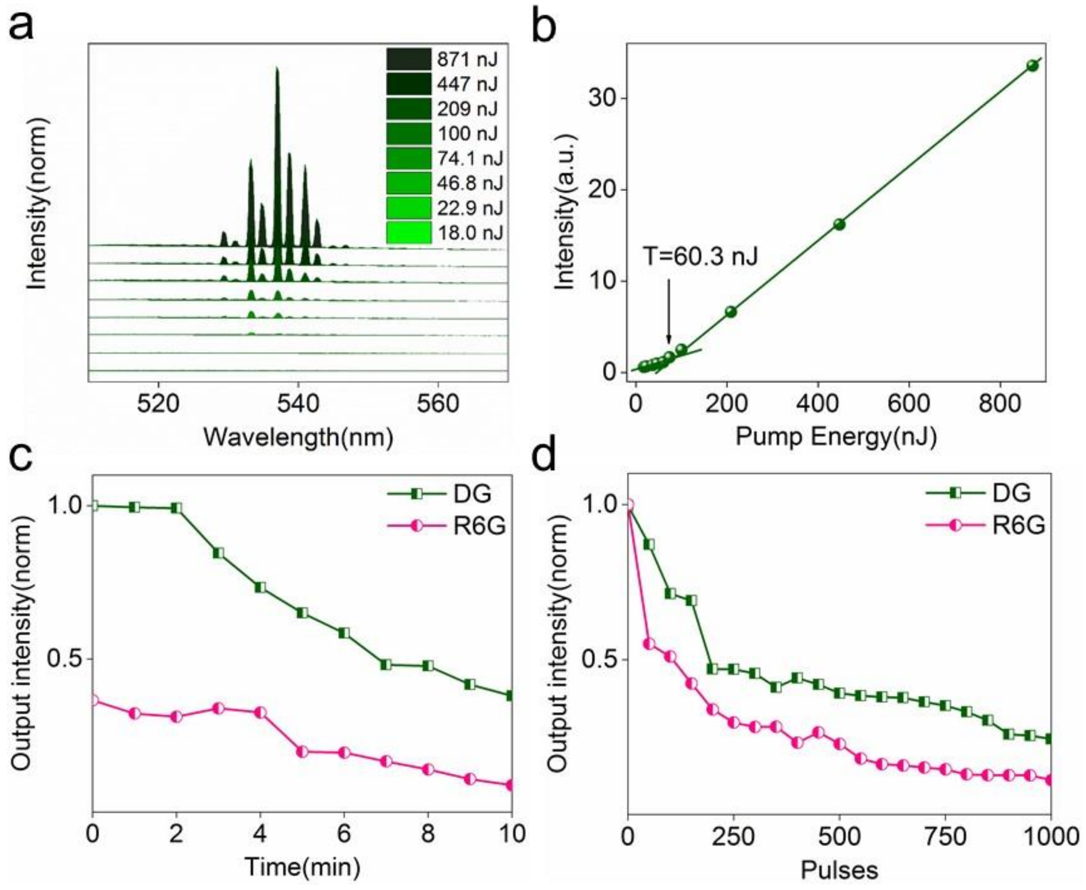

Fig. S12. Laser emission of a DG micro-bead within a cell. a, Lasing output for increased pumping energy. A cell harboring a DG micro-bead was pumped with a 473nm pulsed laser using the optical set-up as shown in Fig. 1a. **b**, After overcoming the lasing threshold, lasing emission depends linearly on the pump energy. The intracellular lasing threshold is 60.3nJ, which is higher than that of a DG bead in pure water (cf. Fig. S1). Experiments were conducted 3 times and data shown are representative. **c &d**, The intracellular lasing intensity of DG microsphere gradually decreases for longer pump durations and increasing number of pulses, which might be caused by photo bleaching.

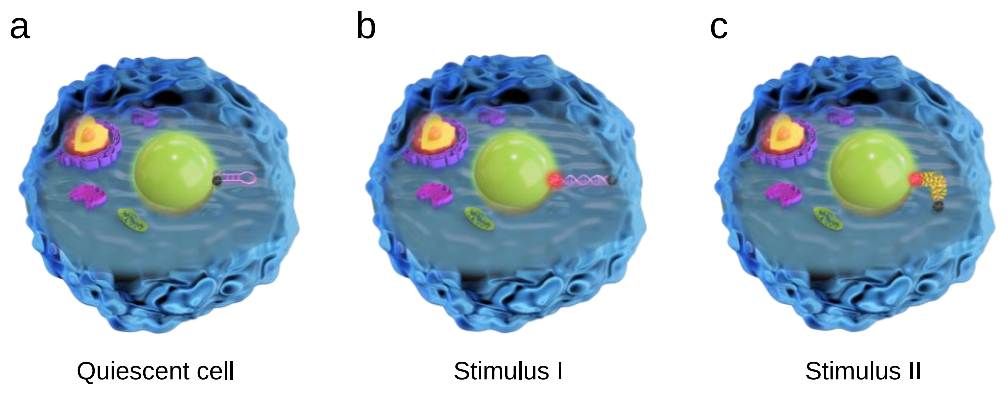

Fig. S13. Schematic illustration of FRET-WGM microcavity for intracellular sensing. a, A cell harboring a FRET-donor doped microsphere whose surface is modified with a DNA probe. Here, for clear illustration, only one DNA molecule is shown. In reality, the DNA probe should cover the whole surface of the microsphere. The DNA probe has a secondary structure, one end is labeled with the FRET-acceptor molecule (grey dot), and the other end is modified with a quencher. At this stage, the FRET acceptor is quenched, so there is not energy transfer. **b**, Intracellular miRNA (or DNA) sensing. When the cell is under type I stimulation, intracellular miRNA level is elevated, which can be specifically recognized by the probe and form a complex. Upon interaction, the secondary structure of the probe distorts and the quencher molecule separates from the FRET-acceptor. As a result, the FRET acceptor fluoresces. If the cell harboring the functionalized microsphere is pumped, the intracellular FRET pair will lase. The difference of resonant wavelength shifts between donor and acceptor ($\Delta\lambda=\lambda_a-\lambda_d$) can be a quantitative readout of the intracellular miRNA molecules. **c**, Intracellular protein sensing. Similarly, when the cell is under type II stimulation, intracellular protein (yellow) level elevates and is specifically recognized by the DNA probe. The protein will form a DNA/protein complex with the probe, which distorts the secondary structure of DNA probe, leading to quencher molecule distanced from FRET-acceptor and thus acceptor molecules fluoresce. If the cell is pumped, the intracellular FRET pair will lase. The difference of resonant wavelength shifts between donor and acceptor ($\Delta\lambda=\lambda_a-\lambda_d$) can be a quantitative readout of the intracellular miRNA molecules.

2. Supplementary tables:

Table S1. Measured FSRs of DG and R6G at various concentrations.

	10nM	50nM	100nM	500nM	10µM	25µM	50µM	75µM	100µM
DG-FSR (nm)	3.803	3.715	3.713	3.429	3.675	3.771	3.443	3.682	3.636
R6G-FSR (nm)	4.367	3.981	3.931	3.881	4.144	4.241	4.141	4.417	4.511

Table S2 Cavity sizes (D) of DG and R6G.

DG microspheres were embedded in different concentrations of R6G solution. A 473 nm ns-pulsed laser was used as a pumping source. At least three microspheres were excited for each R6G concentration. Free spectral range (FSR) of DG and R6G were measured from the obtained spectra. Cavity sizes were then calculated using equation FSR=$\lambda^2/n\pi D$.

R6G (μM)	500			100			50			10			5			1		
Bead No.	1	2	3	1	2	3	1	2	3	1	2	3	1	2	3	1	2	3
Cavity size DG (D_{DG} μm)	15.28	15.82	14.83	15.43	15.26	14.71	15.1	15.11	14.72	14.31	15.17	14.58	14.95	14.71	15.03	14.98	15.05	14.88
Cavity size R6G (D_{R6G} μm)	15.33	16.00	15.01	15.67	15.31	14.94	15.22	15.2	14.76	14.47	15.3	14.97	14.99	14.99	15.05	15.05	15.06	14.96
D_{R6G} - D_{DG}	0.05	0.18	0.18	0.24	0.05	0.23	0.12	0.09	0.04	0.16	0.13	0.39	0.04	0.28	0.02	0.07	0.01	0.08

Table S3. Mathematical models for the nonlinear fitting curves of $\Delta\lambda$ variation with increasing analyte concentration in Fig. 3c and Fig. 4d, and the related parameter values.

	DG-R6G	DG-R6GH
Model	Hill	Hill
Equation	*y=V*x^n/(k^n+x^n)*	*y=V*x^n/(k^n+x^n)*
V	77.37203 ± 7.13189	6.45197 ± 0.11172
k	33.60391 ± 20.75342	6.153.05 ± 0.50413
n	0.3357 ± 0.02892	1.10718 ± 0.06241
Reduced Chi-Sqr	2.36076	0.51104
R square (COD)	0.99397	0.99885
adj. R square	0.9931	0.99839

Table S4. Refractive index measured by an Abbe refractometer

R6G concentration	Refractive index (*n*)	Δn (n-n_0)
0 (H_2O)	1.332	
10nM	1.3351	0.0029
100nM	1.3354	0.0032
10μM	1.3361	0.0039

R6GH concentration		
0 (90%H_2O+10%EtOH)	1.3346	
10nM	1.3367	0.0021
100nM	1.3375	0.0029
10μM	1.3382	0.0036

note: R6G and R6GH are dissolved in H2O and 90%H_2O+10%EtOH, respectively.

3. Theoretical model of FRET-WGM

The molecular processes between donor and acceptor in FRET are illustrated by the Jablonski diagram [1] (Fig. S14 below). When donor molecules (DG) in the microcavity are pumped by a laser, photons transit from the ground state of the donor S_{0d} to the excited state S_{1d}. When all conditions for FRET to occur are met, the excited DG molecules transfer part of their energy to the acceptor molecules (R6G), which results in R6G emission, i.e. energy transition occurs from the excited state of the acceptor S_{1a} to the ground state S_{0a}.

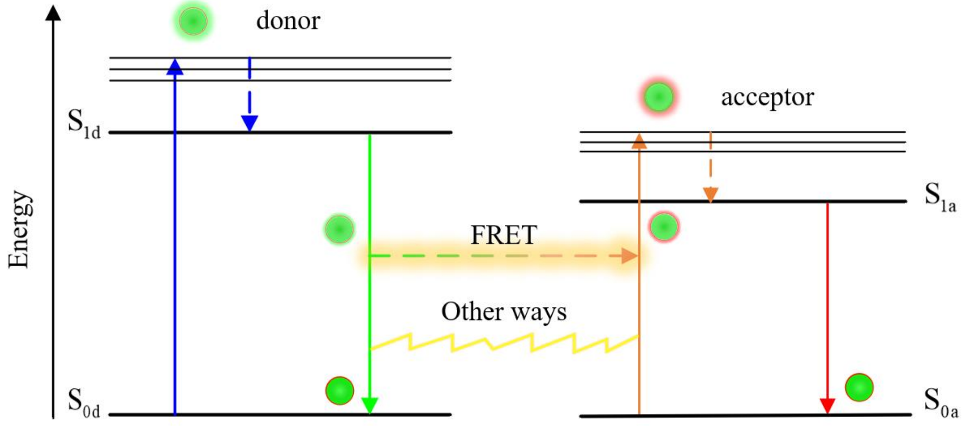

Fig.S14. Energy transfer from donor molecule to acceptor molecule.

The coupled differential equations used to describe the dynamics of the excited-state molecule density and the photon density of the FRET donor-acceptor pairs are as follows:

$$\frac{dn_d(t)}{dt} = I_p(t)[N_d - n_d(t)]\sigma_{pd} + \frac{cq_d(t)}{n_1}\sigma_{add}[N_d - n_d(t)] - \frac{cq_d(t)}{n_1}\sigma_{edd}n_d(t) - k_F n_d(t) - \frac{n_d(t)}{\tau_d} \quad (1)$$

$$\frac{dq_d(t)}{dt} = \frac{c}{n_1 V}\sigma_{edd}n_d(t) + \frac{cq_d(t)}{n_1}\sigma_{edd}n_d(t) - \frac{cq_d(t)}{n_1}\sigma_{add}[N_d - n_d(t)] - \frac{q_d(t)}{\tau_{ed}} + \frac{cq_d(t)}{n_2}\sigma_{ead}n_a(t) - \frac{cq_d(t)}{n_1}\sigma_{aad}[N_a - n_a(t)] \quad (2)$$

$$\frac{dn_a(t)}{dt} = I_p(t)[N_a - n_a(t)]\sigma_{pa} + \frac{cq_a(t)}{n_2}\sigma_{aaa}[N_a - n_a(t)] - \frac{cq_a(t)}{n_2}\sigma_{eaa}n_a(t) + k_F n_d(t) - \frac{n_a(t)}{\tau_a} + \frac{cq_d(t)}{n_1}\sigma_{aad}[N_a - n_a(t)] - \frac{cq_d(t)}{n_2}\sigma_{ead}n_a(t) \quad (3)$$

$$\frac{dq_a(t)}{dt} = \frac{c}{n_2 V}\sigma_{eaa}n_d(t) + \frac{cq_d(t)}{n_2}\sigma_{eaa}n_a(t) - \frac{cq_a(t)}{n_2}\sigma_{aaa}[N_a - n_a(t)] - \frac{q_a(t)}{\tau_{ea}} \quad (4)$$

In which, $n_d(t)$, $n_a(t)$, $q_d(t)$, and $q_a(t)$ represent molecular densities of donor and

acceptor in the excited state, and emitted photon densities of donor and acceptor , respectively. N_d and N_e are the total number of donor and acceptor dye molecules, respectively. σ_{pd} and σ_{pa}, respectively, describe the absorption cross sections of the donor and acceptor molecules at the excitation wavelength. σ_{edd}, σ_{add}, σ_{ead} and σ_{aad} are the donor emission, donor absorption, acceptor emission and acceptor absorption cross sections, respectively, at the donor lasing wavelength. σ_{aaa} and σ_{eaa} are the acceptor emission and acceptor absorption cross sections, respectively, at the acceptor lasing wavelength. $I_p(t)$ is the time-dependent pump intensity and k_F is the FRET rate. n_1 and n_2 represent the resonant cavity and surrounding medium refractive index, respectively. V is the mode volume, and τ_d, τ_a, τ_{ed} and τ_{ea} denote the fluorescence lifetimes of the donor and acceptor, and the cavity decay times at the excitation wavelength of donor and acceptor, respectively.

In equation (1) and (3), the first terms on the right-hand side indicate the number of donors or acceptors that are directly excited by a laser pump from the ground state. The second term designates at the donor excitation wavelength: the number of donors or acceptors at the ground state level that are excited by absorbing the photon energy emitted by the donor were derived based on the spontaneous emission in lasing mode [2-4]. The third terms represent at the donor or acceptor excitation wavelength the reduced numerical densities of the excited state donor or acceptor when the donor or acceptor transitions from the excited state to the ground state through stimulated radiation. The fourth terms are the most critical ones, representing the decrease in the numerical densities of excited state donor and the increase in the numerical densities of excited state acceptor caused by fluorescence resonance energy transfer. The fifth terms indicate that the numerical densities of donor and acceptor in the excited state are reduced due to spontaneous decay. For equation (3), the last two terms represent that, at the donor excitation wavelength, the numerical density of the excited state acceptor increases when the acceptor transitions from the ground state to the excited state by absorbing a photon emitted by the donor and the numerical density of the excited state

acceptor decreases when the acceptor transitions from the excited state to the ground state through stimulated radiation, respectively.

In equations (2) and (4), the first terms on the right-hand side are derived based on the spontaneous emission of the excited donor or acceptor molecules [2-4]. The second terms indicate that, at the corresponding excitation wavelength, the numerical densities of photons emitted when the excited state donor or acceptor transitions to the ground state energy level through stimulated radiation are increased. The third terms designate the reduced numerical densities of photons, when the donor or acceptor transitions from the ground state to the excited state energy level by absorbing the photon energy emitted by the donor or acceptor. The fourth terms represent that the numerical densities of photons emitted by the donor or acceptor are reduced due to the cavity decay times. For equation (2), the last two terms, respectively, indicate, at the donor excitation wavelength, the increased numerical densities of photons when the excited state acceptor transitions to the ground state through stimulated radiation, and the reduced numerical densities of photons, when the donor transitions to the excited state by absorbing the photon energy emitted by the donor.

For the FRET-WGM system, the observed FRET efficiency is high. We obtained a FRET efficiency as high as 58% ($E_{FRET}=(\tau_D-\tau_{DA})/\tau_{DA}$)[5], which is much higher than what was reported in the past [6-9]. The high FRET efficiency implies that the energy transferred to R6G, the acceptor molecule, via FRET alone is sufficient to achieve population inversion, that is to say, FRET dominates the process for R6G lasing. With FRET being the dominating factor, the mechanisms of the active-mode WGM system investigated here are fundamentally different from the mechanisms underlying a non-FRET-WGM system, where the refractive index change is the dominating factor influencing the resonance wavelength.

In the case of a pure WGM (i.e. a non-FRET-WGM) system, as documented in many publications, the shift of the resonance wavelength mainly depends on the refractive index change. For the investigated R6G concentrations, the change of the refractive

index is small (c.f. table S3), corresponding to a small shift of the resonance wavelength $\Delta\lambda$. This explains the much lower sensitivity of a pure WGM compared to a FRET-WGM.

4. Quantitative analysis of wavelength gap ($\Delta\lambda$)—concentration relationship

The acceptor-based laser rate equation is shown in Equation (5):

$$\frac{dq_a(t)}{dt}=\frac{cq_a(t)n_a}{n_2}\sigma_{ea}(\lambda)-\frac{cq_a(t)(N_a-n_a)}{n_2}\sigma_{aa}(\lambda)-\frac{q_a(t)}{\tau_{ea}} \tag{5}$$

Where N_a is the total concentration of acceptor molecules, and n_a is molecular density of acceptor dyes in the excited state. $\sigma_{ea}(\lambda)$ and $\sigma_{aa}(\lambda)$, respectively, are the acceptor emission and absorption cross-section. q_a is the acceptor emitted photon density. τ_{ea} is the lifetime of the photons in the cavity, which equals to $\eta Q\lambda/2\pi c$. n_2 and c represent the refractive index of the surrounding medium (~1.335) and light speed in the vacuum, respectively. η means the fraction of mode energy in the evanescent field. Under steady-state conditions, Equation (5) can be written to Equation (6):

$$n_a\sigma_{ea}(\lambda)-(N_a-n_a)\sigma_{aa}(\lambda)-\frac{2\pi n_2}{\eta Q\lambda}=0 \tag{6}$$

Therefore, from Eqn. (6), we can obtain the fraction of acceptor molecules at the excited state under the threshold condition as described in Equation (7) [10]:

$$\gamma_{tha}=\frac{n_a}{N_a}=\frac{1}{\sigma_{ea}(\lambda)+\sigma_{aa}(\lambda)}[\sigma_{aa}(\lambda)+\frac{2\pi n_2}{\eta Q\lambda N_a}] \tag{7}$$

Where γ_{tha} represents the lasing threshold of acceptor dye.

Similarly, the donor-based laser rate equation can be described in Equation (8).

$$\frac{dq_d(t)}{dt}=\frac{cq_d(t)n_d}{n_1}\sigma_{ed}(\lambda)-\frac{cq_d(t)(N_d-n_d)}{n_1}\sigma_{ad}(\lambda)-\frac{q_d(t)}{\tau_{ed}}+\frac{cq_d(t)n_a}{n_2}\sigma_{ea}(\lambda)-\frac{cq_d(t)(N_a-n_a)}{n_1}\sigma_{aa}(\lambda) \tag{8}$$

Where N_d is the total concentration of donor molecules, and n_d is molecular density of donor dyes in the excited state. $\sigma_{ed}(\lambda)$ *and* $\sigma_{ad}(\lambda)$, respectively, are the donor emission and absorption cross-section. q_d is the donor emitted photon density. τ_{ed} is the lifetime of the photons in the cavity, which equals to $\eta Q\lambda/2\pi c$. n_1 and c represent the refractive index of the microcavity (~1.59) and light speed in the vacuum, respectively. η means the fraction of mode energy in the evanescent field. Under steady-state conditions, Equation (8) can be written to Equation (9):

$$n_d\sigma_{ed}(\lambda)-(N_d-n_d)\sigma_{ad}(\lambda)-\frac{2\pi n_1}{\eta Q\lambda}+\frac{n_1 n_a}{n_2}\sigma_{ea}(\lambda)-(N_a-n_a)\sigma_{aa}(\lambda)=0 \tag{9}$$

From Equation (9), we can obtain the fraction of donor molecules at the excited state under the threshold condition as describe in Equation (10):

$$\gamma_{thd}=\frac{n_d}{N_d}=\frac{1}{\sigma_{ed}(\lambda)+\sigma_{ad}(\lambda)}[\sigma_{ad}(\lambda)+\frac{2\pi n_1}{\eta Q\lambda N_d}+\frac{(1-\gamma_{tha})\sigma_{aa}(\lambda)N_a}{N_d}-\frac{\gamma_{tha}N_a n_1\sigma_{ea}(\lambda)}{N_d n_2}] \quad (10)$$

Where γ_{thd} represents the lasing threshold of the donor dye.

According to Equation (7) and (10), different R6G acceptor concentrations will give to corresponding γ_{th} values of donor or acceptor at respective wavelengths. So when pump energy reaches the threshold (γ_{th}), donor or acceptor will lase at a particular wavelength. Since the absorption and emission cross sections are determined by the dye concentration as well as the energy gain or loss via FRET, also, the emission and absorption cross sections are a continuous function of wavelength, thus, the thresholds will be a function of the wavelength. Therefore, under different acceptor concentrations that energy transfer is different, the laser threshold and resonance wavelength will be different, i.e., the threshold and resonance wavelength are dependent on the acceptor concentration. Therefore, the concentration-dependent shifts of the resonance wavelengths of both donor and acceptor can be simulated. The relationship between concentration and wavelength gap can then be constructed.

To this end, we measured absorption/emission cross-sections of DG and R6G at three R6G concentrations of 50μM, 100μM and 300μM using 2% DG doped microcavity, and calculated the wavelengths and lasing thresholds of DG and R6G using the above equations. The equations used for cross-section calculation are Equation (11) and (12):

$$\sigma_a(\lambda)=3.8\times10^{-21}\varepsilon(\lambda) \quad (11)$$

where ε is the extinction coefficient of R6G and DG. Therefore, the absorption cross-section of R6G and DG can be calculated.

$$\sigma_e(\lambda)=\frac{\lambda^4\mathbf{E}(\lambda)}{8\pi cn^2\tau_F} \quad (12)$$

where c is the speed of light in vacuum, $\mathbf{E}(\lambda)$ is the fluorescence quantum distribution of DG and R6G, τ_F is the fluorescence lifetime (3.6 ns for R6G and 4.1 ns for DG), n is the surrounding medium effective refractive index.

The absorption/emission cross-sections of DG and R6G are shown in Fig. S15a and b.

Using Equation (7), we obtained emission wavelengths of R6G at three different concentrations are 568nm, 571nm and 577nm, respectively, as shown in Fig. S15c &d; whereas the measured CWL data of R6G in the MS are 568.1989nm，571.7778nm and

576.1856nm, respectively, (c.f. Fig. S9d & e). The calculated and experimental wavelengths are in perfect agreement. The calculated data show increased thresholds (Fig. S15c) as concentration increases, which is also consistent with the experimental results presented in the MS (c.f. Figure 4f in MS, please note that data in Fig.4f was obtained using 1%DG doped microcavity).

Using Equation (10), we obtained emission wavelengths of DG at three different concentrations are 523nm, 522nm and 520nm, respectively, as shown in Fig.S15e & f. Whereas the measured CWL data of DG in the MS are 522.9724nm, 521.8584nm and 520.7775nm, respectively, (c.f. Fig. S9d & e). The calculated and experimental wavelengths are also in perfect agreement. In addition, the calculated data show increased thresholds as concentration increases (Fig.S15e), which is also consistent with the experimental results presented in the MS (Figure 4f in MS).

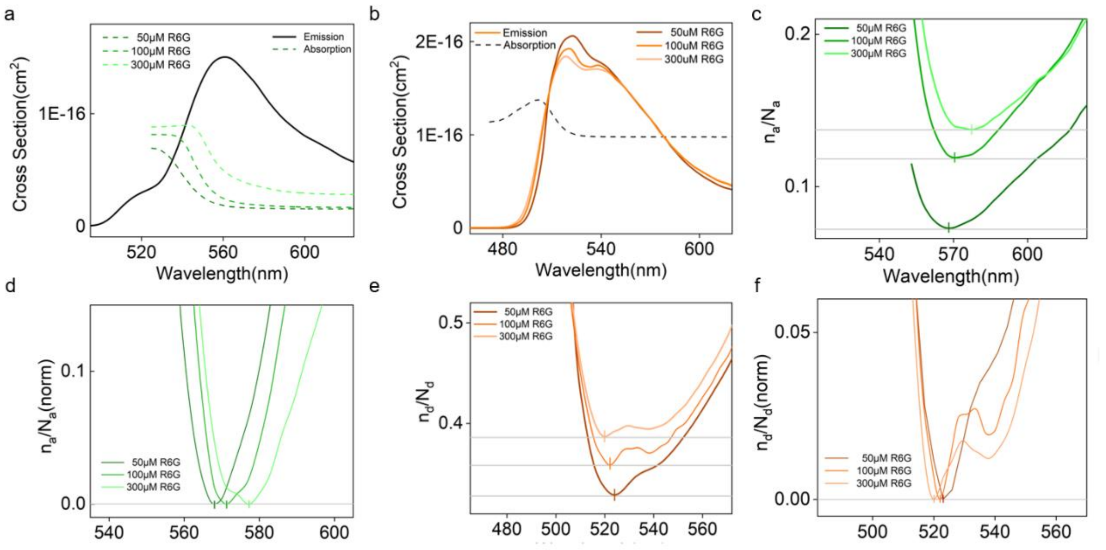

Fig. S15. The theoretical values of DG and R6G emission/absorption cross-sections, wavelength shifts and lasing thresholds. (a) The emission cross-section (solid curve) and the absorption cross-section curves of R6G. (b) The emission cross-section (solid curves) and the absorption cross-section curves of DG. (c) The calculated lasing thresholds and wavelengths of R6G at different concentrations. (d) Normalized laser wavelengths of R6G at different concentration clearly shows redshift as concentration increases. (e) The calculated lasing thresholds and wavelengths of DG at different R6G concentrations. (f) Normalized laser wavelengths of DG at different concentrations clearly shows blue shift as concentration increases.

More importantly, the theoretical prediction, on the one hand, proves the reliability of our experimental data, it also verified the rationality of using the center wavelength (CWL) as a measure in the experiments.

In summary, using our theoretical model, we predicted the donor and acceptor wavelengths shift and lasing thresholds as a function of acceptor concentration. The predicted data perfectly match our experimental results, which, on the one hand, suggests the validity of the theoretical model; also proves the reliability of our experimental data.

Finally, we would like to address that this will be the first report to describe the simultaneous wavelength shifts of donor and acceptor pair in FRET upon coupling with WGM, and reveal the underlined mechanisms in full detail. We are very confident with our data.

5. Supplementary methods

R6GH synthesis

R6GH was synthesized following the recipe in reference 49. To a R6G solution (2.40 g, 5.0 mmol) in 60mL ethanol, 8.0mL (excess) hydrazine monohydrate (80%) was added dropwise under vigorous stirring at room temperature. Then the mixture was heated to reflux in an air bath for 2 h. The mixture was then cooled down and the solvent was removed under reduced pressure. 1M HCl was added to the flask generating a transparent red solution. After that, 1M NaOH was added slowly while stirring until the pH of the solution reached 9–10. The resulting precipitate was filtered and washed three times with 35mL EtOH/water. The crude product was purified by silica gel column chromatography using petroleum ether (b.p. 60–90 ◦C)/ethyl acetate (1:1, *v/v*) as eluent. The molecular weight of the purified product was confirmed by a mass spectrometer (Agilent 1290 Infinity/6460 LC/QQQ MS, ESI ion source; positive ion mode).

Cell membrane stain and laser scanning confocal microscopy (LSCM) analysis

Breast cancer cells were cultured on a PDL coverslip in a 35mm petri dish (5×10^5 cells per dish) for 24h to allow the cells to adhere. Then, the sample was washed three times with PBS, and subsequently, the R6G solution was added to the cells and left for

5 min. Then, all coverslips were quickly submerged in the CellMask^{TM} Deep Red plasma membrane stain solution for 10 minutes at 37 °C. The excessive staining reagent was removed by washing. The cells were fixed with 4% paraformaldehyde and incubated for 10 min at 37°C. After that they were rinsed with PBS buffer, and the coverslips were mounted. The samples were imaged on a laser scanning confocal platform (Leica TCS SP8). The samples were excited by sequentially scanned continuous-wave lasers with a wavelength of 405 nm, 488 nm and 633 nm, respectively. The confocal microscopy data were processed with the Leica Application Suite AF software.

3D confocal imaging (SI Fig. S11a and supplementary video) was performed on a Leica TCS SP8 laser scanning microscope with 20×, 40×, and 63× oil immersion objectives. The Z range was defined from 0 to 20μm and the step distance was set to 0.2μm. The Z-stack acquisition was completed via excitation of the sequentially scanned continuous wave lasers with a wavelength of 405 nm, 488 nm, and 633 nm, respectively.

Time-resolved fluorescence decay

For time-resolved measurements, a TCS-SP8 confocal laser-scanning microscope (Leica) equipped with a 470 nm laser (PDL 800-B, PicoQuant) and time-correlated single photon counting (TCSPC) module Pico Harp 300 (PicoQuant) was used to measure the fluorescence lifetime. A 470 nm pulsed laser was used to excite the fluorescence of DG. The pulse repetition rate of the laser was set at 20 MHz. The fluorescence was captured with an HC PL APO 63× 1.40 NA oil CS2 objective (Leica) with zoom factor of 1 in 128 × 128-pixel format and pinhole size of 5 airy unit at 100 Hz scanning speed (scanning duration was set to 30 s) through a 500–700 nm bandpass filter. The laser power was controlled so that the photon count rate became about 1% of the pulse count rate (2×10^7 Hz). The fluorescence lifetime images were obtained using SymPhoTime's “FAST FLIM” software (PicoQuant). For quantitative analysis of the florescence lifetime, the obtained fluorescence decay curve was fitted with a single exponential function using SymPhoTime software (PicoQuant) using the following

Equation (13):

$$I(t) = A_1 \exp(-t/\tau_1) + A_2 \exp(-t/\tau_2) \qquad (13)$$

Then, the fluorescence lifetime of DG as a FRET donor (τ_D) was calculated using the following Equation (14):

$$\tau_D = (A_1\tau_1^2 + A_2\tau_2^2)/(A_1\tau_1 + A_2\tau_2) \qquad (14)$$

The temporal IRF was 600ps, which after deconvolution resulted in a time resolution of 100 ps.

Statistical analyses and software

Each experiment was repeated at least three times. The data were processed using the Origin 9.0 software. Data are presented as mean values +/- SD

6. References:

[1]. Jablonski, A. Efficiency of Anti-Stokes Fluorescence in Dyes. Nature 131, 839–840 (1933).

[2]. Hebling J, Seres J, Bor Z, et al. Dye laser pulse shortening and stabilization by Q-switching[J]. Optical & Quantum Electronics, 1990, 22(4):375-384.

[3]. Atkinson J, Pace F. The spectral linewidth of a flashlamp-pumped dye laser[J]. IEEE Journal of Quantum Electronics, 1973, 9(6):569-574.

[4]. Aas M, Chen Q, Jonas A, et al. Optofluidic FRET Lasers and Their Applications in Novel Photonic Devices and Biochemical Sensing[J]. IEEE Journal of Selected Topics in Quantum Electronics, 2015, 22(4):188-202.

[5]. Shopova, S.I., Cupps, J.M., Zhang, P., Henderson, E.P. & Fan, X.D. Opto-fluidic ring resonator lasers based on highly efficient resonant energy transfer. Optics Express, 15(20), 12735-42 (2007).

[6]. Chakraborty U, Maiti P, Singha T, et al. Effect of montmorillonite clay on the fluorescence resonance energy transfer between two cationic dyes Acridine Orange and Rhodamine B in solution[J]. Materials today: proceedings, 2020.

[7]. Chakraborty S, Arshad Hussain S. Fluorescence resonance energy transfer (FRET) between acriflavine and CdTe quantum dot[J]. Materials Today: Proceedings, 2020.

[8]. Zambrana-Puyalto X, Ponzellini P, Nicolò Maccaferri, et al. Förster-Resonance Energy Transfer between Diffusing Molecules and a Functionalized Plasmonic Nanopore[J]. Physical Review Applied, 2020.

[9]. Pramanik A, Biswas S, Sekhar Tiwary C, et al. Forster resonance energy transfer assisted white light generation and luminescence tuning in a colloidal graphene

quantum dot-dye system[J]. Journal of Colloid and Interface Science. *565*, 326-336. 2020
[10] Chen, Y.-C.; Chen, Q.; Fan, X., Optofluidic chlorophyll lasers. Lab Chip 2016, 16 (12), 2228-2235.
